# Longitudinal monitoring of mRNA levels of regulatory T cell biomarkers by using non-invasive strategies to predict outcome in renal transplantation

**DOI:** 10.1186/s12882-021-02608-3

**Published:** 2022-02-02

**Authors:** Angelica Canossi, Samuele Iesari, Quirino Lai, Simone Ciavatta, Tiziana Del Beato, Alessandra Panarese, Barbara Binda, Alessandra Tessitore, Franco Papola, Francesco Pisani

**Affiliations:** 1 CNR Institute for Translational Pharmacology, Via Giosuè Carducci 32C, 67100 L’Aquila, Italy; 2 Department of Biotechnological and Applied Clinical Sciences, University of L’Aquila, Via Vetoio, Coppito 2, 67100 L’Aquila, Italy; 3 Pôle de Chirurgie Expérimentale et Transplantation, Institut de Recherche Expérimentale et Clinique, Université catholique de Louvain, Avenue Hippocrate 55, 1200 Brussels, Belgium; 4 Hepatobiliary and Organ Transplantation Unit, Sapienza University of Rome, Viale del Policlinico 155, 00161 Rome, Italy; 5 Regional Center for Organ Transplantation (CRT), S. Salvatore Hospital, Via Lorenzo Natali 1, 67100 L’Aquila, Italy; 6 Regional Centre of Immunohematology and Tissue Typing, San Salvatore Hospital, Via Lorenzo Natali 1, 67100 L’Aquila, Italy

**Keywords:** CTLA-4, FOXP3, RT-PCR, Kidney transplantation, Cellular acute rejection, DSA development, Graft dysfunction

## Abstract

**Background:**

Acute T-cell mediated rejection (aTCMR) is still an issue in kidney transplantation, for it is associated with chronic rejection, graft loss, and overall worse outcomes. For these reasons, a standard non-invasive molecular tool to detect is desirable to offer a simpler monitoring of kidney transplant recipients (KTRs). The purpose of our study was to examine, in peripheral blood before and after transplantation, the expression patterns of regulatory T cell (Treg)-related genes: the forkhead box P3 (FOXP3) and the two CTLA-4 isoforms (full-length and soluble) to predict acute rejection onset, de novo donor-specific antibodies (DSA) development and renal dysfunction 1 year after transplantation.

**Methods:**

We profiled by using a relative quantification analysis (qRT-PCR) circulating mRNA levels of these biomarkers in peripheral blood of 89 KTRs within the first post-transplant year (at baseline and 15, 60 and 365 days, and when possible at the acute rejection) and compared also the results with 24 healthy controls.

**Results:**

The three mRNA levels drastically reduced 15 days after transplantation and gradually recovered at 1 year in comparison with baseline, with very low levels at the time of aTCMR for FOXP3 (RQ = 0.445, IQR = 0.086–1.264, *p* = 0.040), maybe for the pro-apoptotic role of FOXP3 during inflammation. A multivariate Cox regression analysis evidenced a significant relation between aTCMR onset and thymoglobuline induction (HR = 6.749 *p* = 0.041), everolimus use (HR = 7.017, *p* = 0.007) and an increased risk from the solCTLA-4 expression at 15 days, mainly considering recipients treated with Mycophelolic acid (HR = 13.94 *p* = 0.038, 95%CI:1.157–167.87). Besides, solCTLA-4 also predisposed to graft dysfunction (eGFR< 60 mL/min/1.73m^2^) at 1 year (AOR = 3.683, 95%CI = 1.145–11.845, *p* = 0.029). On the other hand, pre-transplant solCTLA-4 levels showed a protective association with de novo DSAs development (HR = 0.189, 95%CI = 0.078–0.459, *p* < 0.001).

**Conclusions:**

mRNA levels of Treg-associated genes, mainly for solCTLA-4, in peripheral blood could put forward as candidate non-invasive biomarkers of cellular and humoral alloreactivity in clinical transplantation and might help shape immunosuppression, tailor monitoring and achieve better long-term outcomes of kidney transplantation in the wake of “precision medicine”.

**Supplementary Information:**

The online version contains supplementary material available at 10.1186/s12882-021-02608-3.

## Background

T-cell mediated acute rejection (aTCMR) is still an issue in kidney transplantation for its association with chronic rejection, graft loss, and overall worse outcomes. In addition, immunosuppressive therapy bears a risk of infection, malignancy and cardiovascular disease. For this reason, tailored immunosuppression strategies are useful to curb adverse events associated with kidney transplantation. Therefore, risk prediction and early diagnosis of aTCMR through non-invasive methods can be crucial for allograft survival and immunosuppression management [1–4]. For these reasons, developing a standard clinical and molecular assessment procedure offers a simpler monitoring of KTRs. The study of biomarkers of aTCMR and immune dysregulation in renal transplantation has progressively focused on regulatory T cells (Tregs). This CD4^+^CD25^+^FOXP3^+^ lymphocytic subpopulation, which spreads from the thymus as effector and memory suppressive cells, is essential in suppressing alloimmune response and maintaining tolerance in transplantation FOXP3 expression is the major determinant of Tregs phenotype and function. Another important marker recently investigated in kidney transplantation and aTCMR is CTLA-4 (CD152). CTLA-4 is implicated in self-tolerance and acts as a braking co-inhibitor of activated CD4^+^ and CD8^+^ T cell responses. Tregs represent the principal cellular population expressing the CTLA-4 [5]. CTLA-4 is encoded by the homonymous gene located on chromosome 2 (2q33) in two transcripts in humans: a transmembrane isoform, resulting from the translation of all 4 exons of the gene (full length CTLA-4, flCTLA-4), and a truncated isoform of exon-3, encoding the transmembrane domain (soluble CTLA-4, solCTLA-4) following alternative splicing. The inhibitory function of CTLA-4 is carried out through several different mechanisms, comprehending the cell-extrinsic action of its *soluble* form responsible for competition with CD28, namely the CTLA-4 counterpart, which transduces instead a proliferation signal for T cells [6–9].

The purpose of our study was to develop a new non-invasive diagnostic tool, based on an accurate analysis of molecular FOXP3 and CTLA-4 mRNA expression pattern in peripheral blood, during the first post-transplant year, capable to predict aTCMR onset, de novo DSA development and renal dysfunction. For doing this, we performed three different analyses: 1) a prospective longitudinal monitoring of mRNA levels of FOXP3, flCTLA-4 and solCTLA-4 during the first post-transplant year; 2) a case-control study of KTRs compared to healthy controls for FOXP3 and 3) an evaluation of diagnostic power of the variables investigated.

## Methods

### Population

One hundred and twenty-five patients consecutively underwent kidney transplantation at the Organ Transplantation Unit of the Regional Hospital of L’Aquila, Italy during the period January 2011–September 2017. Of them, 120 (96%) received a kidney from a deceased-brain donor and five (4%) from a living donor. Written informed consent was obtained from all participants. Investigations were carried out by the rules of the Declaration of Helsinki, and the institutional ethical committee approved the study (protocol no 0098164/2011). Out of the total KTRs, 89 met the inclusion criteria for the study consisting in: a) sufficient/pure mRNA collected at least two of the four sample collection time-points; b) no steroid-resistant rejection. Twenty-four healthy blood donors with comparable age, sex, and ethnicity volunteered as controls (all Caucasian subjects).

KTRs were treated according to the local immunosuppression protocol. Induction therapy was carried out with basiliximab (Simulect®, Novartis, Basel, Switzerland) in 82 cases (92.1%), and with antithymocyte globulins (Thymoglobulin®, Sanofi, Paris, France) in 7 cases (8.9%). Maintenance therapy comprised prednisone, a calcineurin inhibitor (CNI) (either tacrolimus [Advagraf®, Astellas Pharma, Tokyo, Japan], in 75 cases (84.3%), or cyclosporine [Neoral®, Novartis] in 14 cases (15.7%), and a proliferation signal inhibitor. The latter consisted of mycophenolic acid in 77 cases (86.5%), and everolimus (Certican®, Novartis) in 12 cases (13.5%). Patients treated with everolimus were considered separately, given the effect of this drug on an increased risk of aTCMR within the first postoperative year in this analysis.

### Outcomes

Graft function was reported as estimated glomerular filtration rate (eGFR) [10]. When a filtrate rate was < 60 mL/min/1.73m^2^ for 3 months or more, the patient was considered suffering from chronic renal failure.

The diagnosis of acute rejection was biopsy-proven and we considered in the analysis only KTRs with acute T-cell-mediated rejection (aTCMR), classified in the categories 3 and 4 of Banff’15 classification [11]. Rejection treatment was based on three intravenous one-gram methylprednisolone boluses over 3 days.

Screening for HLA antibody was performed by Lambda Cell Tray T cell CDC-based Class-I PRA and Lambda Antigen Tray Mixed Class-I/II ELISA (One Lambda, Canoga Park, CA, USA). Detection of donor specific antibodies (DSA) was performed on T and B lymphocytes by cell-based assays (CDC-XM) and Luminex solid-phase assays. Blood samples were collected at four time points (baseline, post-KTR day 15, 60, 365) and at the time of a possible aTCMR. All patients were followed-up until 2 years minimum.

### RNA isolation and gene expression analysis

Peripheral venous blood (3 ml) was drawn directly into Tempus Blood RNA tubes (Thermo Fisher Scientific Inc., Waltham, MA-US), according to the manufacturer’s protocol, frozen and stored at − 20 °C until processing. Whole blood total RNA was extracted using a Tempus Spin RNA isolation (Thermo Fisher Scientific Inc.), which uses an RNA isolation method (6–25 μg of RNA) with silica columns and an additional DNase treatment. The purity and concentration of this RNA were analysed using an ultraviolet-visible (DU 530 spectrophotometer, Beckman Coulter Life Sciences, Brea, CA-US). One microgram of total isolated RNA was employed for complementary DNA synthesis (cDNA) with the High Capacity cDNA Reverse Transcription kit (Applied Biosystems, Foster City, CA-US).

Gene expression profiles of the three gene targets (FOXP3, flCTLA-4 and solCTLA-4) were analysed through a quantitative real-time (RT)-PCR (qRT-PCR, 2^-∆∆CT^ method) using glyceraldehyde 3-phosphate dehydrogenase (GADPH) and β2 microglobulin (β2M), respectively, as internal control. The relative quantification analysis was estimated by using the cDNA from baseline samples (pre-transplantation) to calibrate all the three transcripts. One control blood sample was also used as reference for FOXP3 RT-PCR quantification.

mRNA expression analysis was performed in duplicate/triplicate using predesigned TaqMan Gene Expression Assays (FOXP3: HS00203958-ml, GAPDH: HS99999905-ML, flCTLA-4: HS01011591-ml, solCTLA-4: HS03044419, β2M: HS00984230; Life Technologies, Monza, Italy) and a standard protocol. RT-PCR amplification was performed in 48-well plates on a StepOne Real Time PCR system (Applied Biosystems, CA, USA) and RQ were calculated using StepOne v.2.3 software for automated data analysis (Applied Biosystems). The comparison between the clinical subgroups of KTRs (i.e. aTCMR-free vs. aTCMR-positive patients) was normalized with the 2^-∆CT^ logarithm 10 (log2^-∆CT^) compared to the endogenous control, to correct the asymmetric distribution of the data.

### Statistical analysis

Binomial variables were reported using numbers and proportions. Numerical variables were reported using means ± standard deviations (SD), or medians and interquartile ranges (IQR), as appropriate. Gene levels distribution is shown as box- or scatter-plot representations. Results were compared using Fisher’s exact test or Mann-Whitney U test/Wilcoxon. Comparison between groups and correlation between variables were examined by parametric (t test/one-way ANOVA, Pearson’s correlation), and non-parametric tests (Kruskal Wallis, Friedman test for repeated measures and Spearman’s test), as appropriate.

Receiver-operating characteristic (ROC) curves were generated for the prediction of short- and long-term aTCMR episodes, de novo DSA development and renal dysfunction after transplantation to define the accuracy of diagnostic test and establish the best cut-off for clinical outcomes.

The predictive ability of several variables for the risk of the acute rejection, graft dysfunction and post-transplant development of DSA in our population was assessed. All factors considered in univariable analyses were based on literature review and suggestions from the clinical team. Logistic regressions were run for simply dichotomous variables. The crude odds ratios (OR), 95% confidence interval (CI) and *p* value were reported for each predictor in the univariable analysis. Only statistically significant variables in the univariable analysis were entered into multiple logistic regression analysis to predict the final independent factors. The model fit was assessed by chi-square, degrees of freedom and *p*-value. We chose a backward conditional method to select significant independent covariates.

We used the Cox proportional hazards model for time-dependent events (graft loss, death, acute rejection, de novo anti-DSA antibody development). All the covariates with *p* ≤ 0.05 were introduced into multivariable models. Hazard ratios (HRs), and 95% confidence intervals (CIs) were reported for significant variables.

The significance of statistical tests was taken at two-tailed *p* < 0.05. Analyses were run with SPSS Statistics v.13.0 (SPSS Inc., Chicago, IL), GraphPad Prism v.6 (GraphPad Software, La Jolla, CA-US), and MedCalc v.19.2.0 (MedCalc Software Ltd., Ostend, Belgium).

## Results

### Characteristics and follow-up of the recipients

Clinical features of the patients (*n* = 89) transplanted between 2011 and 2017 are reported in Table 1. Mean age at transplant was 52.5 ± 11.5 years. All patients had a minimum follow-up of 2 years (mean follow-up period: 38.3 ± 23.8 months).

**Table 1:** Demographic and clinical characteristics of the transplant study groups

Variables	Frequencies
	N (%)
Recipient gender:	
- males	65 (73%)
- females	24 (27%)
Recipient age (years, mean±SD)	52.5±11.5
Donor age (years, mean±SD)	50.9±15.9
Donor gender:	
- males	59 (66%)
- females	30 (34%)
Donor type:	
- brain-dead donors	84 (94.3%)
- living donors	5 (5.7%)
Time on RRT (months, mean±SD)	54.3±35.0
RRT type:	
- haemodialysis	74 (83.1%)
- peritoneal dialysis	15 (16.9%)
No of HLA mismatches (median and IQR)	3MM (1-5)
Class I PRA (%, mean±SD)	4.4±11.1
Class II PRA (%, mean±SD)	1.8±7.8
CIT (minutes, mean±SD)	630±265
WIT (minutes, mean±SD)	44±13
Induction:	
- basiliximab	82 (92%)
- anti-thymocyte globulins (rATG)	7 (8%)
Calcineurin inhibitor:	
- cyclosporine	14 (16%)
- tacrolimus	75 (84%)
Proliferation signal inhibitor:	
- everolimus	12 (13%)
- mycophenolic acid	77 (87%)
CMV-positive donor/CMV-negative recipient	11 (12.4%)
Previous transplantation	6 (6.7%)
Delayed graft function	32 (36%)

Abbreviations: *CIT* cold ischaemia time, *CMV* cytomegalovirus, *HLA* human leukocyte antigen, *IQR *interquartile range, *PRA* panel-reactive antibodies, *RRT* renal replacement therapy, *SD* standard deviation, *WIT* warm ischaemia time

During the entire study period, three patients (3.4%) died: in all the cases, the cause of death was an infection. During the same period, six graft losses (6.7%) were reported. In four cases, an immunological cause was reported, namely a drug-resistant acute rejection and a chronic active antibody-mediated rejection in two cases, respectively. In the remaining two cases, a graft thrombosis and a graft pyelonephritis were reported.

Eighteen patients showed episodes of rejection during the follow-up period, sixteen had cellular or mixed acute rejections (cell and/or antibody-mediated rejections) and two showed chronic rejection episodes (mixed or Ab-mediated).

Seventy-three patients (82.0%) did not develop acute rejection. On the opposite, 16 patients (18.0%) exhibited at least one episode of aTCMR, of whom 11/16 (68.8%) cases during the first year. The median time from transplantation to the first episode of aTCMR was 2.1 (IQR = 0.8–10.6) months in the 11 cases experiencing a rejection within the 1st year from the transplant, while in the entire population was 9.95 (IQR = 1.05–15.7) months.

Twenty-three KTRs in total (25.8%) developed DSA after transplantation, 19 within the first year. In total, 17 (89.5%) cases presented DSAs reactive against HLA-class I, whereas eight (42.1%) against class-II. The eGFR of KTRs in post-transplant period varied from a median value of 42.6 (IQR = 25.1–61.2) ml/min/1.73m^2^ after 15 days, to 50.9 (IQR = 32.1–65.8) at 60 days, 55.2 (IQR = 36.3–72.0) after 1 year from transplantation and 58.1 (39.1–75.7) at last follow-up.

### Characteristics of the healthy controls

Twenty-four healthy blood donors enrolled for the study showed comparable characteristics respect to the population of transplanted patients. In detail, age (52.5 ± 11.5 vs. 52.8 ± 12.5 years, *p* = 0.920), gender (65 males [73.0%] and 24 females [27.0%] vs. 14 males [58.3%] and 10 females [41.7%], *p* = 0.250), and Caucasian ethnicity (89 [100.0%] vs. 24 [100.0%], *p* = 1.000) were not statistically different between KTRs and healthy controls, respectively.

### Longitudinal evaluation of flCTLA-4, solCTLA-4, and FOXP3 mRNAs during the first year after transplantation (RT-PCR reference: pre-transplant)

**Fig. 1:**
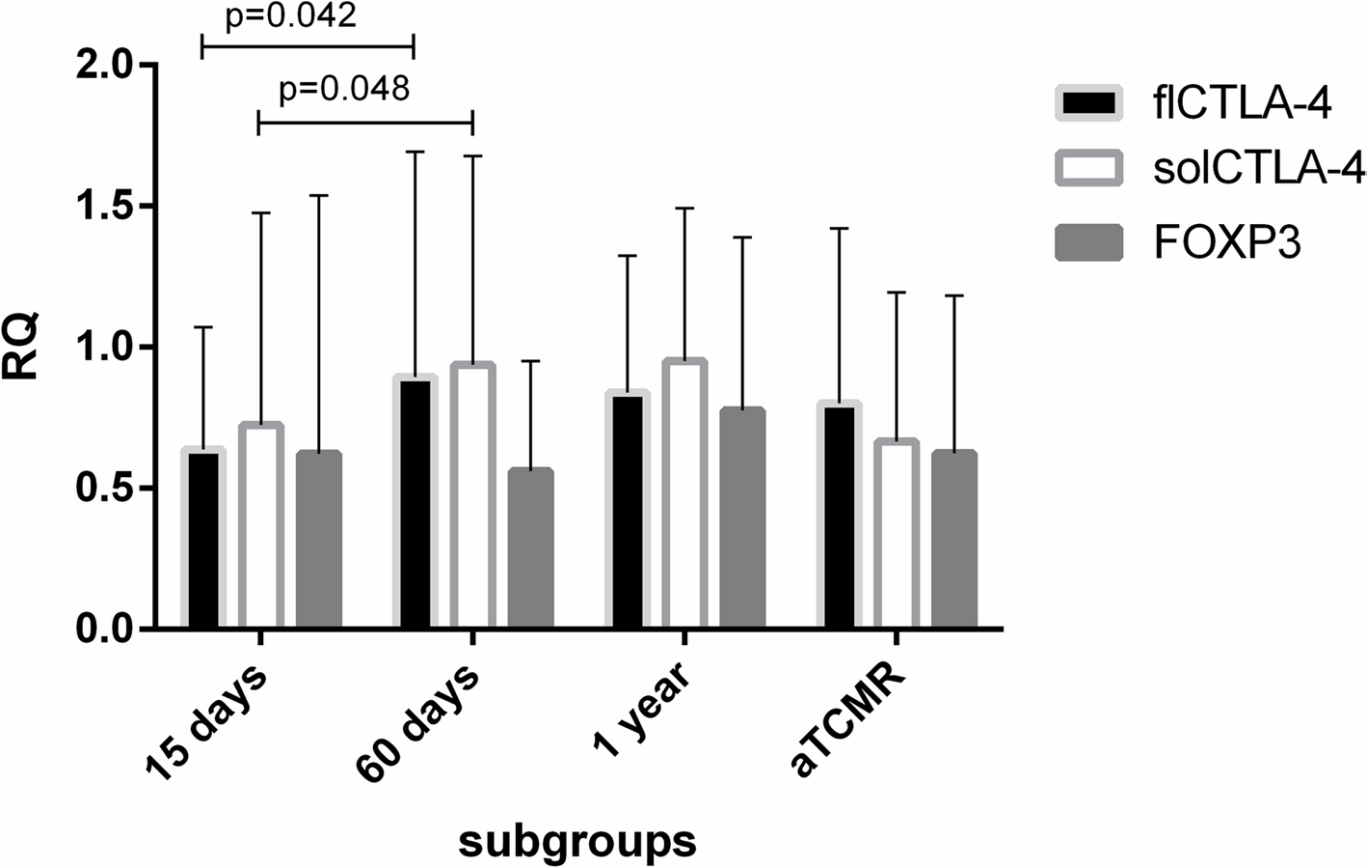
Time-related trend of mRNA levels of flCTLA-4, solCTLA-4, and FOXP3 (mean±SD) in all KTRs in the post-transplant period compared to baseline mRNA values. The expression levels were monitored at 15, 60 and 365 days, and at the moment of the ACR. A significant increase between 15 days and 60 days was highlighted for CTLA-4 isoforms (flCTLA-4 RQ=0.895±0.798, *p=0.042, solCTLA-4 RQ=0.937±0.740, *p=0.048), while FOXP3 slightly decreased. Later every marker reverted to baseline levels. In case of rejection episode, flCTLA-4 expression was the highest compared to the other two molecules at the time of the adverse event

We compared the expression of CTLA-4 isoforms and FOXP3 before and across the first year after transplantation in all the 89 included KTRs compared to baseline. We found a reduction in the expression of all the candidate biomarkers after 15 days (flCTLA-4: RQ = 0.638 ± 0.433; solCTLA-4: RQ = 0.724 ± 0.752; FOXP3: RQ = 0.623 ± 0.915), a significant increase of the expression after 60 days for CTLA-4 isoforms (Wilcoxon signed rank test: flCTLA-4 *p* = 0.042; solCTLA-4 *p* = 0.048) and, on the contrary, a slight decrease for FOXP3 expression (RQ = 0.561 ± 0.391, *p* = 0.991). After 1 year, the relative expression of all three markers partially recovered to baseline levels. In case of rejection episode, flCTLA-4 expression was the highest compared to the other two molecules at the time of the adverse event (flCTLA-4: RQ = 0.800 ± 0.620, solCTLA-4: RQ = 0.666 ± 0.529, FOXP3: RQ = 0.624 ± 0.559, Kruskal Wallis test: *p* = 0.763, Fig. 1).

### Longitudinal evaluation of FOXP3 expression levels depending on the type of maintenance immunosuppression (RT-PCR reference: healthy controls)

The trend of FOXP3 mRNA levels over time up to 1 year after transplantation on all KTRs compared to healthy controls followed that observed for CTLA-4 isoforms and, by distinguishing the KTRs for immunosuppressive regimen, we observed that there were no differences between recipients treated with everolimus and recipients on mycophenolic acid for the whole duration of the monitoring (Additional file 1: Fig. S1).

### Case-control study of FOXP3 mRNA expression between KTRs and healthy controls

**Fig. 2:**
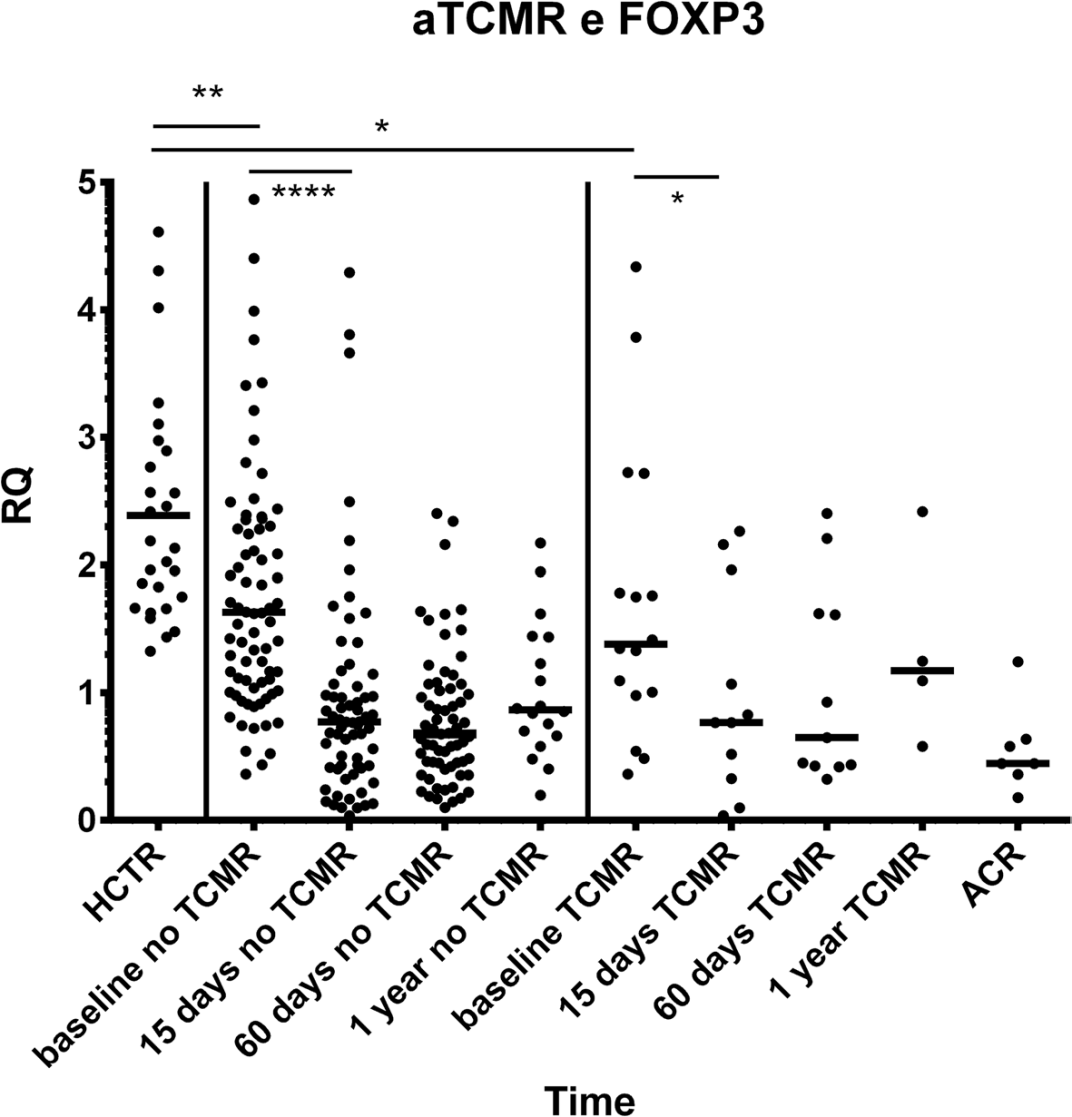
Comparison of the median RQs of FOXP3 mRNA between healthy controls (HCR) and recipients. Recipients are divided into two subgroups of KTRs experiencing or not aTCMR. Controls represent the reference for RT-PCR. **Controls vs. baseline ACR-free KTRs: RQ=2.132, IQR=1.664-2.895 vs. RQ=1.630, IQR=1.072-2.367, p=0.005; and *ACR-positive KTRs: RQ=1.381, IQR=0.986-2.483, *p*=0.010. **** Controls vs. 15-day ACR-free KTRs: RQ=0.771, IQR=0.415-1.169 and ACR-positive KTRs: RQ=0.767, IQR=0.327-1.962, Wilcoxon signed rank *p*<0.001 and *p*=0.0002, respectively. The expression of FOXP3 at the time of ACR was lowest compared to baseline (RQ=0.445, IQR=0.360-0.636, p=0.035). The choice of the FOXP3 molecule as tolerance marker candidate was supported by evidence of a wide inter-individual variability in both healthy (min RQ: 1.326 - max: 4.612) and dialysis patients (min RQ: 0.362- max: 7.968). Abbreviations: ACR, acute cellular rejection; (fl- or sol)CTLA-4, (full-length or soluble) cytotoxic T lymphocyte-4 antigen; FOXP3, forkhead box P3; KTR, kidney transplant recipient; RQ, relative quantification; RT-PCR, real-time polymerase-chain reaction; ds, days; yr, year

FOXP3 expression profiles showed differences between KTRs and healthy controls. Controls had higher levels of mRNA compared to KTRs at baseline (controls vs. aTCMR-free cases: median RQ = 2.132 vs. 1.630, *p* = 0.005; vs. aTCMR-positive cases: RQ = 1.381, *p* = 0.010). Distinguishing between the aTCMR-free and -positive patients, despite of limited cohort, we evidenced a similar trend between two groups of patients, with an initial significant reduction in FOXP3 levels at 15 days after transplantation and a gradual enhancement of expression up to 1 year, but always below baseline levels (Wilkoxon signed rank test *p* < 0.001 and *p* = 0.015), Friedman ANOVA test *p* < 0.001. The expression of FOXP3 at the time of acute rejection was the lowest when compared to baseline (median RQ = 0.445, vs. aTCMR-free baseline RQ = 1.630, *p* = 0.040 or aTCMR-positive RQ = 1.381, paired t test *p* = 0.035, Fig. 2).

### Association between aTCMR onset and clinical or molecular variables

**Fig. 3:**
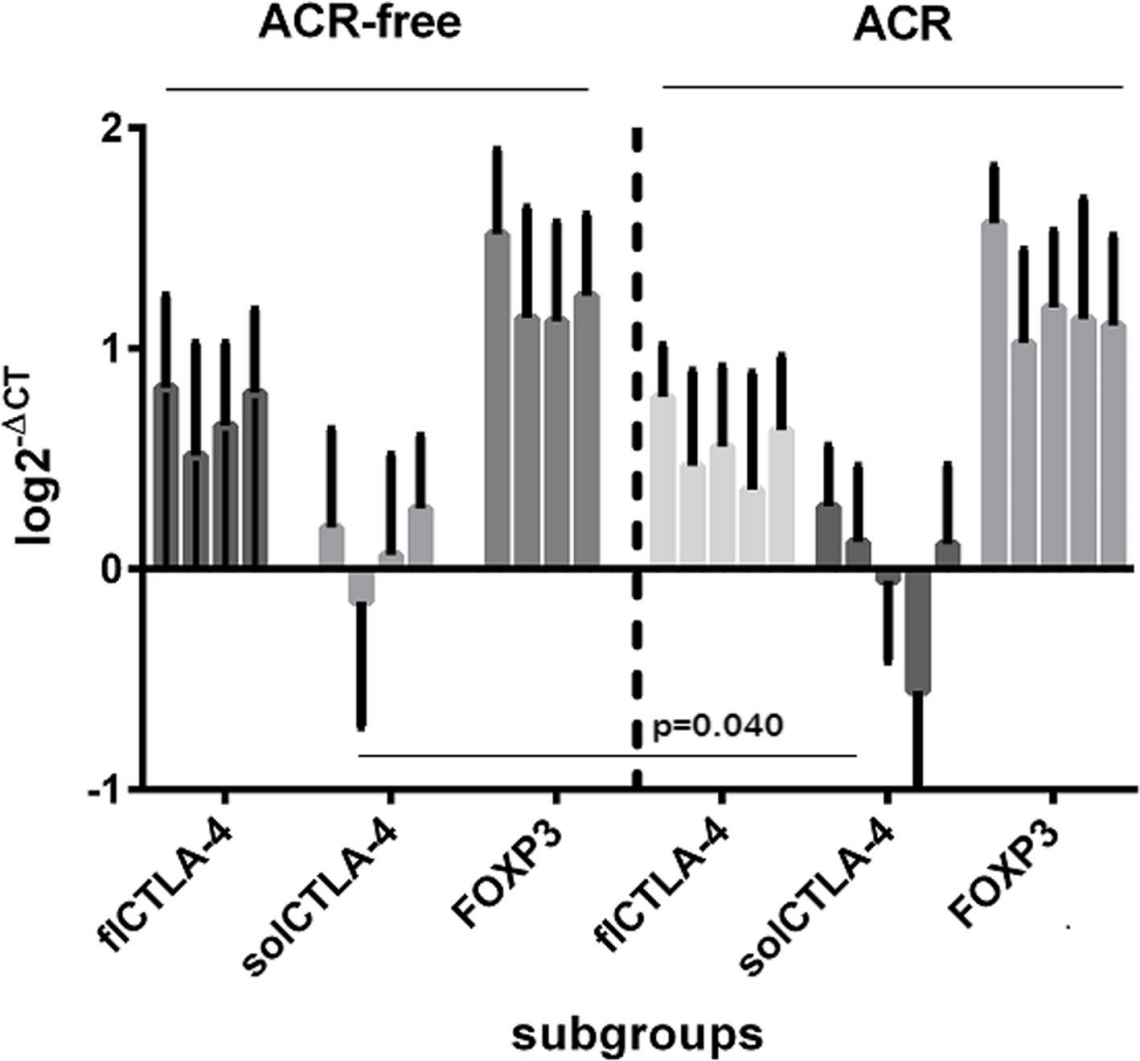
Profile of the transcript levels (log2-ΔCT) of the three molecular markers in the two groups of aTCMR-free and aTCMR-positive KTRs (mean±SD) in comparison to pre-transplant values. Histogram log values: baseline, 15 days, 60 days, one year and rejection time. There were no significant differences between KTRs with and without TCMR within 1 year after transplantation concerning FOXP3 and the flCTLA-4 isoforms. Only solCTLA-4 levels showed a different trend after transplantation, with a significant higher transcript levels fifteen days after transplantation in cases with aTCMR (log=0.365, IQR=-0.073-0.648, vs. -0.070, IQR=-0.440-0.280, *p*=0.04)

We examined several clinical variables along with the molecular targets to assess their predictive ability for the cumulative risk of short- and long-term aTCMR after transplantation. There were no significant differences between KTRs with and without aTCMR within 1 year after transplantation concerning FOXP3 and the flCTLA-4 isoforms (Fig. 3). Only solCTLA-4 levels showed a different trend after transplantation, with significant higher transcript levels 15 days after transplantation in cases with aTCMR, compared to aTCMR-free KTRs (log = 0.365, IQR = -0.073–0.648, vs. -0.070, IQR = -0.440–0.280, *p* = 0.040). Furthermore, some clinical data influenced the onset of aTCMR, such as the use of everolimus (aTCMR = 29.4% vs. aTCMR-free = 9.7%, *p* = 0.048, OR = 3.869, 95%CI = 1.051–14.231), induction type (protection of basiliximab: 72.7% vs. 94.9%, *p* = 0.038 OR = 0.144), HLA-MMs (3.8 ± 1.4 vs. 3.0 ± 1.2, *p* = 0.050), immunosuppression switch (54.5% vs. 16.7%, *p* = 0.010, OR = 6.000), as reported in Table 2.

**Table 2:** Comparison of immunological biomarkers (log 2^-DCT^), demographic and clinical parameters in the two groups of patients with aTCMR within 1 year (*n* = 11) or stable transplant (*n* = 78)

VARIABLE	aTCMR (*n* = 11)	aTCMR-free (*n* = 78)	*P* value	OR=
^a^FOXP3, baseline	1.580 (1.410-1.670)	1.585 (1.370-1.760)	0.909	
^a^FOXP3, 15d	1.150 (0.718-1.675)	1.260 (0.950-1,510)	0.999	
^a^FOXP3, 60d	1.345 (1.003-1.648)	1.180 (0,940-1,390)	0.284	
^a^FOXP3, 1y	1.320 (1.030-1.420)	1.350 (1,095-1,423)	0.776	
^a^flCTLA-4 baseline	0.910 (0.730-0.980)	0.810(0.600-1.008)	0.586	
^a^flCTLA-4 15d	0.655 (0.010-1.093)	0.570 (0.340-0.740)	0.691	
^a^flCTLA-4 60d	0.585 (0.320-1.020)	0.630 (0.440-0.870)	0.999	
^a^flCTLA-4 1y	0.870 (0.740-1.260)	0.730 (0.508-0.863)	0.210	
^a^solCTLA-4 baseline	0.320 (0.060-0.590)	0.235 (0.035-0.443)	0.376	
^a^**solCTLA-4 15d**	0.365 (-0.073-0.648)	-0.070 (-0.440-0.280)	**0.043**	
^a^solCTLA-4 60d	0.120 (-0.170-0.230)	0.155 (-0.190-0.363)	0.539	
^a^solCTLA4 1y	0.070 (-0.530-0.670)	0.110 (0.060-0.500)	0.999	
Recipient gender (%):				
-males	8/11 (72.7)	57/78 (73.1)	1.000	
-females	3/11 (27.3)	21/78 (26.9)		
Donor gender (%):				
-males	6/11 (54.5)	53/78 (67.9)	0.498	
-females	5/11 (45.5)	25/78 (32.1)		
^b^Recipient age	54.7±14.3	52.2±11.1	0.372	
^b^Donor age	51.8±20.4	50.8±15.3	0.747	
Donor type (%):				
-brain-dead donors	11/11 (100)	73/78 (93.6)	1.000	
-living donors	0/11 (0)	5/78 (6.4)		
Delayed graft function (%)	4/11 (36.4)	28/78 (35.9)	1.000	
Induction (%):				
-**Basiliximab**	8/11 (72.7)	74/78 (94.9)	**0.038**	**0.144**
-Anti-thymocyte globulin	3/17 (27.3)	4/78 (5.1)		
Proliferation signal inhibitor (%):				
** -Everolimus**	5/17 (29.4)	7/72 (9.7)	**0.048**	**3.869**
-Mycophenolic acid	12/17 (70.6)	65/72 (90.3)		
Calcineurin inhibitor (%):				
-Cyclosporine	2/11 (18.2)	12/78 (15.4)	0.682	
-Tacrolimus	9/11 (81.8)	66/78 (84.6)		
^b^HLA Mismatches (MM)	3.8 ± 1.4	3.0±1.2	**0.050**	
PRA n= (%)				
-Class I	2/11 (18.2)	34/78 (43.6)	0.188	
-Class II	2/11 (18.2)	25/78 (32.1)	0.493	
^b^Time on RRT (months)	51.5±26.6	54.7±36.1	0.963	
^b^CIT (min)	717.0±218.6	617.9 ±269.4	0.212	
^b^WIT (min)	48.0±16.5	43.4±12.1	0.416	
Previous transplants (%)	1/11 (9.1)	5/78 (6.4)	0.558	
**Maintenance therapy change %**	6/11 (54.5)	13/78 (16.7)	**0.010**	**6.000**
CMV reactivation (%)	5/11 (45.5)	17/78 (21.8)	0.131	

^a ^(median ± interquartiles), ^b ^(mean ± SD)

**Fig. 4:**
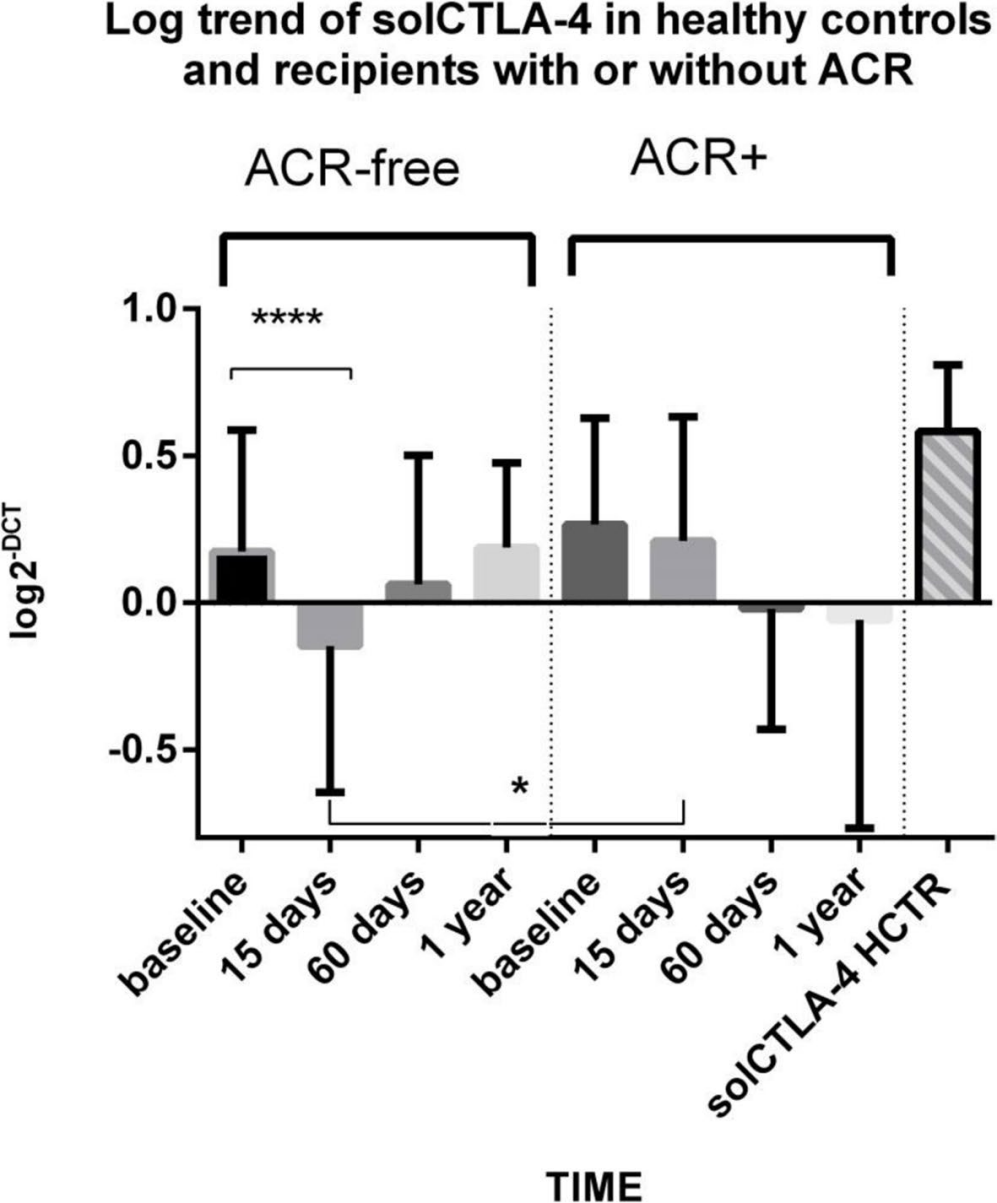
Profile of solCTLA-4 transcripts (mean ±SD) as a function of acute cellular rejection in patients receiving mycophenolic acid. * ACR KTRs: 0.210±0.127 vs. ACR-free KTRs: -0.147±0.07, *p*=0.013, at 15 days after transplantation. ****Baseline ACR-free KTRs: 0.174±0.052 vs. 15 days: -0.147±0.069, *p*<0.001. Healthy controls: 0.582±0.047. The solCTLA-4 expression in the group of patients without rejection was significantly different among various time points, ANOVA Friedman test: *p*<0.001

**Fig. 5:**
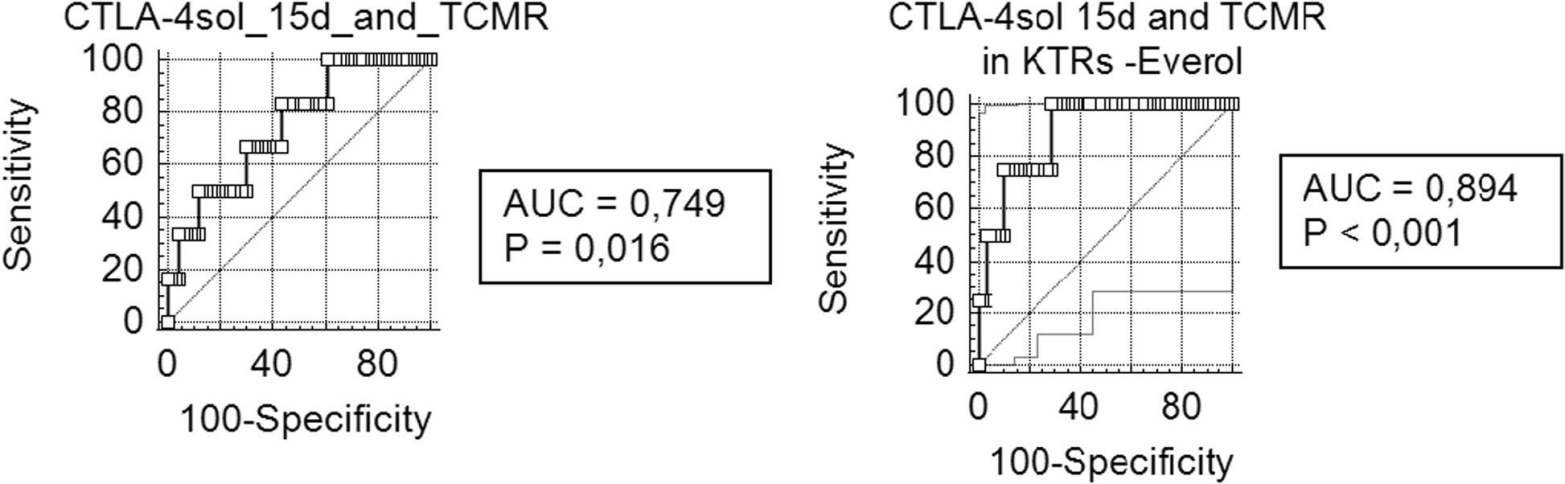
Receiver-operating characteristic (ROC) curve analyses for the expression of the CTLA-4sol at day 15. Prediction of aTCMR episodes within the 1st year in the whole cohort of patients (stable transplantation n=67, acute cellular rejection episodes n=6) and in those receiving only mycophenolic acid (n=63). 1) AUC=0.749, p=0.016 95%CI=0.634-0.843. Younden’s index threshold showed for solCTLA-4 log criterion values > -0.05 a prediction of acute rejection with sensitivity of 83.3% and specificity of 56.7%. 2) AUC=0.894, p<0.000 95%CI=0.791-0.953. solCTLA-4 15 days, Younden’s criterion for values > 0.13 a prediction of acute rejection with sensitivity of 100.0% and specificity of 71.2%

We evaluated then the time-dependent risk for aTCMR of molecular targets and clinical parameters by using univariate and multivariate Cox regression analyses and we found out a significant positive relation with thymoglobuline induction (HR = 6.749 *p* = 0.041) and everolimus use (HR = 7.017, *p* = 0.007) and a trend to increased risk from the solCTLA-4 expression at 15 days (HR = 3.905, *p* = 0.057, Table 3). Considering only recipients treated with Mycophelolic acid (Fig. 4), the risk for aTCMR of solCTLA-4 at 15d was significantly predictive for the time-dependent risk (*p* = 0.038 HR = 13.94 95%CI: 1.157–167.87). The receiver operating characteristic curve (ROC) analysis confirmed that 15-day solCTLA-4 showed good diagnostic ability of aTCMR (AUC = 0.749, 95%CI:0.634–0.843 *p* = 0.020) considering the whole KTR population and, with the exclusion of patients treated with everolimus, displayed this molecule like a more accurate biomarker for acute rejection (AUC = 0.894, 95% CI:0.791–0.957, *p* < 0.001 Fig. 5). A cut-off value > 0.13 predicted aTCMR with a sensitivity of 100.0% and a specificity of 71.2%

**Table 3:** Univariate and multivariate Cox regression analyses for the risk of aTCMR after kidney transplantation

VARIABLE	Univariate analysis			Multivariate analysis^a^		
				-2ln likelihood: 46.938		
	HR	95% CI	P	HR	95% CI	P
Baseline						
Membrane CTLA4	1.178	0.366—3.788	0.784	—	—	—
Soluble CTLA4	2.382	0.744—7.624	0.144			
FOXP3	0.691	0.196—2.433	0.565	—	—	—
At 15 days						
Membrane CTLA4	1.053	0.321—3.457	0.932	—	—	—
**Soluble CTLA4**	3.164	0.836—11.981	0.090	**3.905**	**0.958-15.916**	**0.057**
FOXP3	0.698	0.202—2.408	0.569	—	—	—
At 60 days						
Membrane CTLA4	2.537	0.480—13.404	0.273	—	—	—
**Soluble CTLA4**	1.431	0.362—5.661	0.609	—	—	—
FOXP3	4.343	0.868—21.715	0.074	—	—	—
At 365 days						
Membrane CTLA4	1.898	0.160—22.480	0.611	—	—	—
Soluble CTLA4	0.711	0.056—8.955	0.792	—	—	—
FOXP3	0.749	0.095—5.895	0.783	—	—	—
Recipient age	1.007	0.963—1.053	0.758	—	—	—
Donor age	0.999	0.969—1.031	0.972	—	—	—
Recipient gender	1.128	0.363—3.506	0.835	—	—	—
Donor gender	0.930	0.337—2.567	0.889	—	—	—
Type of dialysis	0.575	0.185—1.790	0.340	—	—	—
Dialysis duration	0.752	0.398—1.424	0.382	—	—	—
CMV reactivation	2.033	0.738—5.604	0.170	—	—	—
Previous transplant	1.466	0.973—2.210	0.067	—	—	—
HLA mismatch	1.229	0.885—1.707	0.219	—	—	—
CIT (min)	0.799	0.251—2.544	0.704	—	—	—
WIT (min)	1.000	0.998—1.002	0.859	—	—	—
Type of donor	1.030	0.992—1.069	0.120	—	—	—
**Type of induction **	**0.259**	0.082—0.814	**0.021**	**0.148**	**0.024-0.923**	**0.041**
Chronic use of steroids	0.667	0.213—2.093	0.488	—	—	—
CNI (tacrolimus vs. cyclosporine)	23.310	0.011-51229.762	0.423	—	—	—
**PSI (mTOR vs. mycophenolate)**	**3.926**	1.425—10.813	**0.008**	**7.017**	**1.714-28.725**	**0.007**
**Immunosuppression change**	**9.175**	3.178—26.488	**0.000**	—	—	—
Infratherapeutic CNI level at 15 days	0.821	0.291—2.311	0.708	—	—	—
Infratherapeutic CNI level at 60 days	1.303	0.483—3.514	0.601	—	—	—
DGF	0.729	0.252—2.114	0.561	—	—	—

^a^ Model summary: χ^2^(1)=15.901, *p *< 0.001. Covariates initially introduced in the multivariable model and then elided were: soluble CTLA4 at 15 days, FOXP3 at 60 days

Abbreviations: *CI* confidence intervals, *CIT* cold ischemia time, *CMV* cytomegalovirus, *CNI* calcineurin inhibitor, *DGF* delayed graft function, *HLA* human leukocytes antigens, *HR* hazard ratio, *PRA* panel-reactive antibodies, *mTORi* mammalian target of rapamycin inhibitor, *PSI* proliferation signal inhibitor, *WIT* warm ischemia time

### flCTLA-4, solCTLA-4 and FOXP3 mRNA expression levels in KTRs developing de novo DSA

**Fig. 6:**
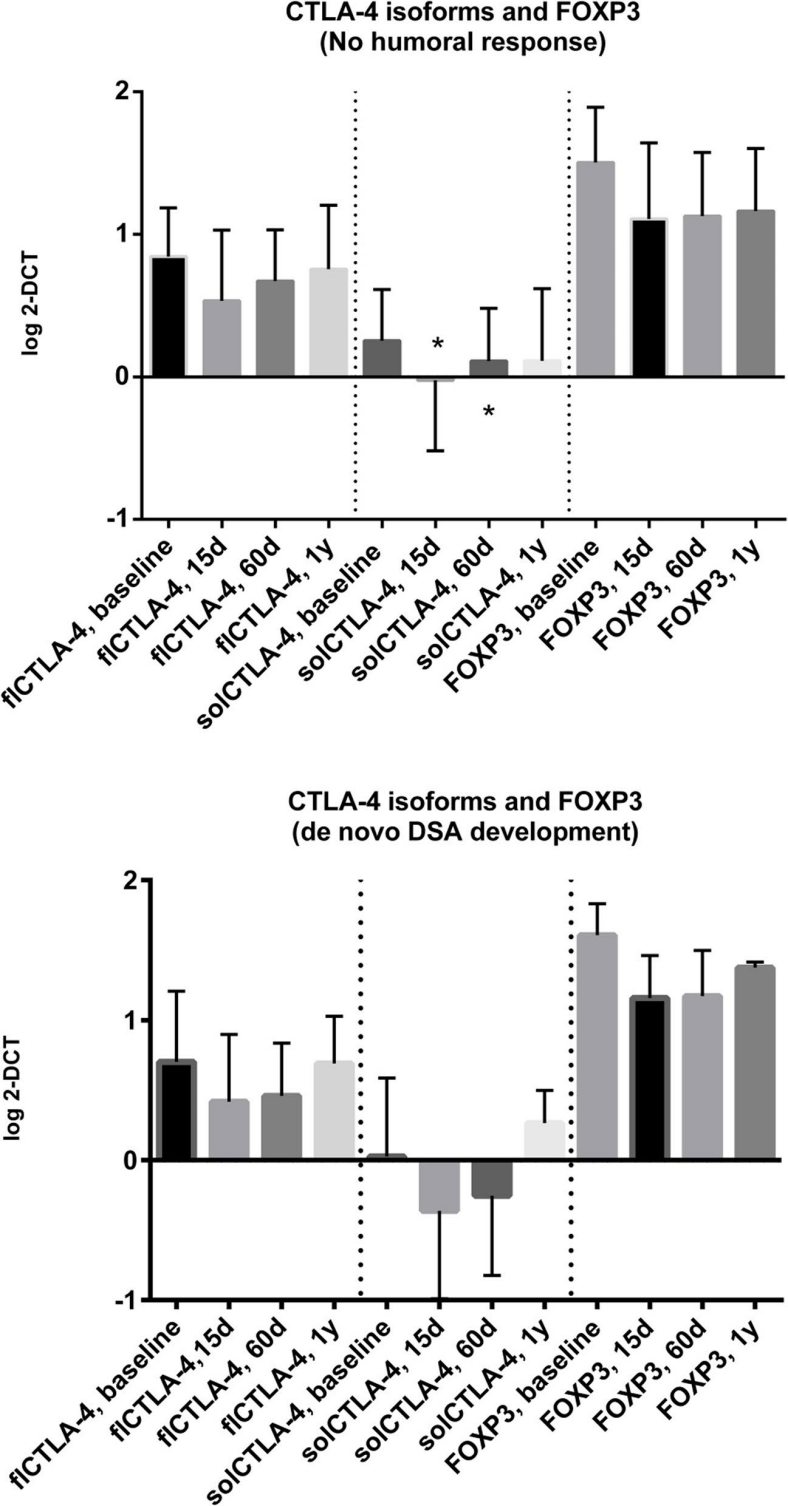
flCTLA-4, solCTLA-4, and FOXP3 mRNA expression levels (mean ±SD) in patients with (bottom) and without (top) de novo DSA. *Day-15 solCTLA-4: DSA+ log= -0.362±0.627 vs. DSA- -0.024±0.495, *p*=0.047; ** Day-60 solCTLA-4: DSA+ log= -0.254±0.570 vs. DSA- =0.110±0.371, *p*=0.020. Abbreviations: (fl- or sol)CTLA-4, (full-length or soluble) cytotoxic T lymphocyte-4 antigen; DSA, donor-specific antibodies; FOXP3, forkhead box P3; KTR, kidney transplant recipient; d, days; y, year

Twenty-three KTRs in total (25.8%) developed DSA after transplantation. By examining the trend of the expression of the three candidate biomarkers up to 1 year in correlation with the de novo DSA development (Fig. 6)**,** we found out a different time-dependent trend for solCTLA-4 molecule between two groups of patients, which decreased after 15 days from transplantation but with significant differences (DSA negative:-0.024 ± 0.495 vs. baseline = 0.254 ± 0.359, *p* < 0.001; DSA positive:-0.362 ± 0.627 vs. baseline = 0.026 ± 0.560, *p* = 0.0005), and then increased at 60 days, significantly in the group without DSA (0.110 ± 0.371, *p* = 0.022). Expression increased at 1 year in both groups. The trend of flCTLA-4 and FOXP3 expression was like solCTLA-4 but it was comparable between the two groups of patients. Pre-transplant FOXP3 levels were significantly different (DSA negative: 1.504 ± 0.388 vs. DSA positive: 1.607 ± 0.225 *p* = 0.033)**.**

At univariable regression, we found that solCTLA-4 was negatively associated with DSA development at baseline (OR = 0.284, 95%CI = 0.085–0.896 *p* = 0.042), 15 days (OR = 0.325, 95%CI = 0.116–0.911 p = 0.033), and at 60 days (OR = 0.167, 95%CI = 0.044–0.636 *p* = 0.009). At multivariable analysis, only *baseline* solCTLA-4 (AOR = 0.110, 95%CI = 0.023–0.537, *p* = 0.006) proved an independent variable, with protective effect on DSA development in the shorter and longer 2 years-term (Additional file 2: Table S1 and Additional file 3: Table S2). At multivariate Cox regression analysis for the risk of de novo DSA development, pre-transplant solCTLA-4 proved to be a time-dependent negative predictor of humoral response (HR = 0.189, 95%CI = 0.078–0.459, *p* < 0.001, Table 4).

**Table 4:** Univariate and multivariate Cox regression analyses for the risk of development of de novo donor-specific antibodies after kidney transplantation (*p* < 0.05)

VARIABLE	Univariate analysis			Multivariate analysis^a^		
				-2ln likelihood: 103.840		
	HR	95% CI	P	HR	95% CI	P
Baseline						
Membrane CTLA4	0.479	0.181—1.263	0.137	—	—	—
**Soluble CTLA4**	0.296	0.126—0.694	**0.005**	**0.189**	0.078-0.459	**<0.001**
FOXP3	1.603	0.467—5.501	0.453	—	—	—
At 15 days						
Membrane CTLA4	0.810	0.352—1.862	0.619	—	—	—
**Soluble CTLA4**	0.399	0.196—0.810	**0.011**	—	—	—
FOXP3	1.494	0.579—3.851	0.406	—	—	—
At 60 days						
Membrane CTLA4	0.413	0.120—1.427	0.162	—	—	—
**Soluble CTLA4**	0.205	0.074—0.563	**0.002**	—	—	—
FOXP3	1.194	0.370—3.854	0.767	—	—	—
At 365 days						
Membrane CTLA4	0.969	0.216—4.356	0.967	—	—	—
Soluble CTLA4	1.570	0.350—7.045	0.556	—	—	—
FOXP3	1.826	0.304—10.972	0.510	—	—	—
Recipient age	0.975	0.943—1.008	0.132	—	—	—
Donor age	1.003	0.977—1.029	0.847	—	—	—
Recipient gender	1.240	0.460—3.341	0.671	—	—	—
Donor gender	0.507	0.224—1.151	0.104	—	—	—
Type of dialysis	1.378	0.408—4.659	0.606	—	—	—
Dialysis duration	1.005	0.994—1.016	0.383	—	—	—
Don CMV IgG+/Rec CMV IgG-	1.201	0.355—4.060	0.768	—	—	—
Previous transplant	0.676	0.091—5.025	0.702	—	—	—
HLA mismatch	0.956	0.694—1.318	0.785	—	—	—
CIT (min)	0.999	0.998—1.001	0.411	—	—	—
WIT (min)	0.999	0.969—1.031	0.960	—	—	—
Type of donor	3.235	0.949—11.025	0.061	—	—	—
Type of induction	1.321	0.308—5.659	0.708	—	—	—
Chronic use of steroids	1.366	0.183—10.183	0.761	—	—	—
CNI (tacrolimus vs. cyclosporine)	0.743	0.218—2.529	0.635	—	—	—
PSI (mycophenolate vs. mTOR)	1.550	0.527—4.562	0.426	—	—	—
Immunosuppression change	1.330	0.524—3.376	0.549	—	—	—
Infratherapeutic CNI level at 15 days	1.020	0.430—2.424	0.964	—	—	—
Infratherapeutic CNI level at 60 days	1.002	0.442—2.275	0.996	—	—	—
DGF	0.958	0.406—2.263	0.922	—	—	—
CMV reactivation	0.605	0.206—1.780	0.361	—	—	—

^a^ Model summary: χ^2^(1)=14.217, *p* < 0.01. Covariates initially introduced in the multivariable model and then elided were: soluble CTLA4 at 15 days, soluble CTLA4 at 60 days

Abbreviations: *CI* confidence intervals, *CIT* cold ischemia time, *CMV* cytomegalovirus, *CNI* calcineurin inhibitor, *DGF* delayed graft function, *HLA* human leukocytes antigens, *HR* hazard ratio, *PRA* panel-reactive antibodies, *mTORi* mammalian target of rapamycin inhibitor, *PSI* proliferation signal inhibitor, *WIT* warm ischemia time

### Correlations between FOXP3 and CTLA-4 isoforms with graft dysfunction after transplantation

Examining differences in biomarkers mRNA expression between positive- and negative-graft dysfunction KTRs, we recorded a positive correlation between post-transplant graft dysfunction (eGFR< 60 ml/min/1.73m^2^) and baseline FOXP3 levels, (median = 1.590, IQR = 1.408–1.823 vs. 1.520, IQR = 1.295–1.678, *p* = 0.030), or 15-day solCTLA-4 (− 0.050, IQR = -0.225–0.340 vs. -0.190, IQR-0.700-0.260, *p* = 0.039). We detected significant differences between patients with and without graft dysfunction in terms of demographic and clinical parameters (Table 5), such as recipient age (mean = 55.7 ± 10.4 vs. 47.4 ± 11.3 years, *p* < 0.001), donor age (57.9 ± 12.6 vs. 40.6 ± 15.1, *p* < 0.001, and class I PRA+ (28.0% vs. 52.8%, *p* = 0.035), confirmed also by univariable regression. A multivariable analysis revealed that graft dysfunction 1 year after transplantation was independently associated with 15-day solCTLA-4 (AOR = 3.683, 95%CI = 1.145–11.845 *p* = 0.029) and donor age (AOR = 1.084, 95%CI = 1.033–1.137 *p* = 0.001, Table 6), while baseline FOXP3 levels did not reach significance (AOR = 3.012 *p* = 0.100). At 2 years, only donor age remained independently associated (Additional file 4: Table S3).

**Table 5:** Immunological, demographic and clinical parameters in the two groups of patients with or without graft dysfunction (eGFR<60ml/min/1.73m^2^) one year after transplantation

VARIABLE	Graft dysfunction (*N* = 50)	No dysfunction (*N* = 36)	*P* value	OR=
**FOXP3**				
**Baseline**^a^	**1.590 **(1.408-1.823)	**1.520** (1.295-1.678)	**0.033**	
15 days^a^	1.270 (0.999-1.520)	1.125 (0.510-1.435)	0.156	
60 days^a^	1.270 (0.970-1.398)	1.160 (0.940-1.430)	0.952	
One year^a^	1.420 (1.280-1.660)	1.215 (1.015-1.363)	0.055	
**flCTLA-4**				
Baseline^a^	0.850 (0.623-1.060)	0.825(0.580-0.980)	0.713	
15 days^a^	0.610 (0.365-0.845)	0.550 (-0.020-0.740)	0.072	
60 days^a^	0.620 (0.443-0.870)	0.645 (0.285-0.913)	0.763	
One year^a^	0.890 (0.2800-1.290)	0.740 (0.640-0.800)	0.790	
**solCTLA-4**				
Baseline^a^	0.240 (0.105-0.443)	0.280 (-0.125-0.508)	0.457	
**15 days**^a^	**-0.050 (-0.225-0.340)**	**-0.190 (-0.700-0.260)**	**0.039**	
60 days^a^	0.145 (-0.008-0.370)	0.090 (-0.230-0.330)	0.559	
One year^a^	0.500 (-0.06-0.633)	0.100 (0.005--0.318)	0.402	
Recipient gender (M/F)	37/50	25/36	0.825	
Donor gender (M/F)	29/50	27/36	0.161	
**Recipient age**^b^	55.7±10.4	47.4±11.3	**0.0002**	
**Donor age**^b^	57.9±12.6	40.6± 15.1	**<0.0001**	
Induction:				
- Basiliximab (%)	48/50 (96.0)	31/36 (86.1)	0.124	
- Anti-thymocyte globulins (%)	2/50 (4.0)	5/36 (13.9)		
CNI (cyclosporine vs. tacrolimus)	10/50 (20.0)	4/36 (11.1)	0.378	
mTORi (%)	8/50 (16.0)	4/36 (11.1)	0.754	
HLA mismatches^b^	3.1±1.1	3.0±1.5	0.785	
**Class I PRA (%)**	14/50 (28.0)	19/36 (52.8)	**0.035**	
Class II PRA (%)	10/50 (20.0)	14/36 (38.9)	0.092	
Time on dialysis (months)^b^	58.4±36.9	46.4±26.4	0.160	
Cold ischemia time (min)^b^	632.4±294.1	620.2±227.4	0.836	
Warm ischemia time (min)^b^	43.1±14.0	44.8±11.1	0.175	
Previous transplants (%)	5/50 (10.0)	1/36 (2.8)	0.394	
Maintenance therapy change (%)	13/50 (26.0 )	6/36 (16.7)	0.443	
Proteinuria at one year (mg/l)^b^	159.9±180.4	148.2±156.8	0.564	
DSA development (%)	12/50 ( 24.0)	7/36 (19.4)	0.811	
DGF (%)	21/50 (42.0)	10/36 (27.8)	0.260	
CMV reactivation (%)	13/50 (26.0)	8/36 (22.2)	0.457	

^a^ (median ± interquartiles), ^b^ (mean ± SD), ^c^ Mann Whitney U test. Real-time biomarkers values expressed as log 2^∆CT^. Graft dysfunction is defined as an eGFR<60ml/min/1.73m^2^ one year after transplantation

**Table 6:** Univariable and multivariable logistic regression for the risk of graft dysfunction at one year from kidney transplantation

Variables	Univariable analysis			Multivariable analysis^a^		
				-2log likelihood: 69.886		
	OR	95% CI	P	AOR	95% CI	P
**Baseline**						
Full-length CTLA4	2.033	0.643-6.428	0.227	—	—	—
Soluble CTLA4	2.610	0.882-7.729	0.083	—	—	—
FOXP3	3.111	0.869-11.133	0.081	—	—	—
**At 15 days**						
Full-length CTLA4	2.462	0.897-6.752	0.080	—	—	—
***Soluble CTLA4***	**2.628**	1.015-6.800	**0.046**	**3.683**	1.145-11.845	**0.029**
FOXP3	2.301	0.836-6.331	0.107	—	—	—
**At 60 days**						
Full-length CTLA4	1.962	0.519-7.412	0.320	—	—	—
Soluble CTLA4	1.726	0.559-5.328	0.343	—	—	—
FOXP3	1.132	0.376-3.402	0.826	—	—	—
**At one year**						
Full-length CTLA4	1.356	0.163-11.318	0.778	—	—	—
Soluble CTLA4	2.574	0.302-21.913	0.387	—	—	—
FOXP3	4.381	0.261-73.437	0.304	—	—	—
**Recipient age**	1.072	1.026-1.121	**0.002**	—	—	—
Recipient gender	1.095	0.417-2.873	0.854	—	—	—
**Donor age**	1.094	1.049-1.141	**0.000**	**1.084**	1.033-1.137	**0.001**
Donor gender	0.395	0.150-1.036	0.059	—	—	—
Type of donor	1.085	0.172-6.852	0.931	—	—	—
Previous transplantation	3.043	0.326-28.445	0.329	—	—	—
HLA mismatch	1.106	0.787-1.554	0.563	—	—	—
cRF (first class)	0.421	0.174-1.019	0.055	—	—	—
cRF (second class)	0.552	0.220-1.387	0.206	—	—	—
CIT	1.000	0.999-1.002	0.547	—	—	—
WIT	1.004	0.971-1.038	0.820	—	—	—
Type of renal replacement therapy	1.796	0.586-5.502	0.305	—	—	—
Dialysis time	1.001	0.989-1.001	0.889	—	—	—
CMV reactivation	1.230	0.449-3.371	0.688	—	—	—
Type of induction^b^	0.258	0.047-1.415	0.119	—	—	—
CNI Tacrolimus vs. Cyclosporine	0.500	0.143-1.744	0.277	—	—	—
Use of everolimus	1.302	0.351-4.831	0.693	—	—	—
Immunosuppression change	3.111	0.929-10.422	0.066	—	—	—
DGF	2.534	0.965-6.658	0.059	—	—	—
Development of DSA	1.579	0.531-4.699	0.412	—	—	—
Proteinuria at one year (mg/l)	1.002	0.998-1.005	0.344			

^a^Model summary: χ^2^(3)= 25.088, *p* = 0.000; Nagelkerke R^2^ = 0.406; Hosmer and Lemeshow χ^2^ test = 3.715, *p* = 0.882. Covariates initially introduced in the multivariable model and then elided were: recipient age, cRF class I and DGF; ^b^use of anti-thymocyte globulins vs. use of anti-IL2 receptor-α monoclonal antibodies; ^c^ vs. tacrolimus

Abbreviations: *OR* odds ratio, *CI* confidence intervals, *AOR* adjusted OR, *BMI* body mass index, *HLA* human leukocyte antigens, *CIT* cold ischemia time, *WIT* warm ischemia time

**Fig. 7:**
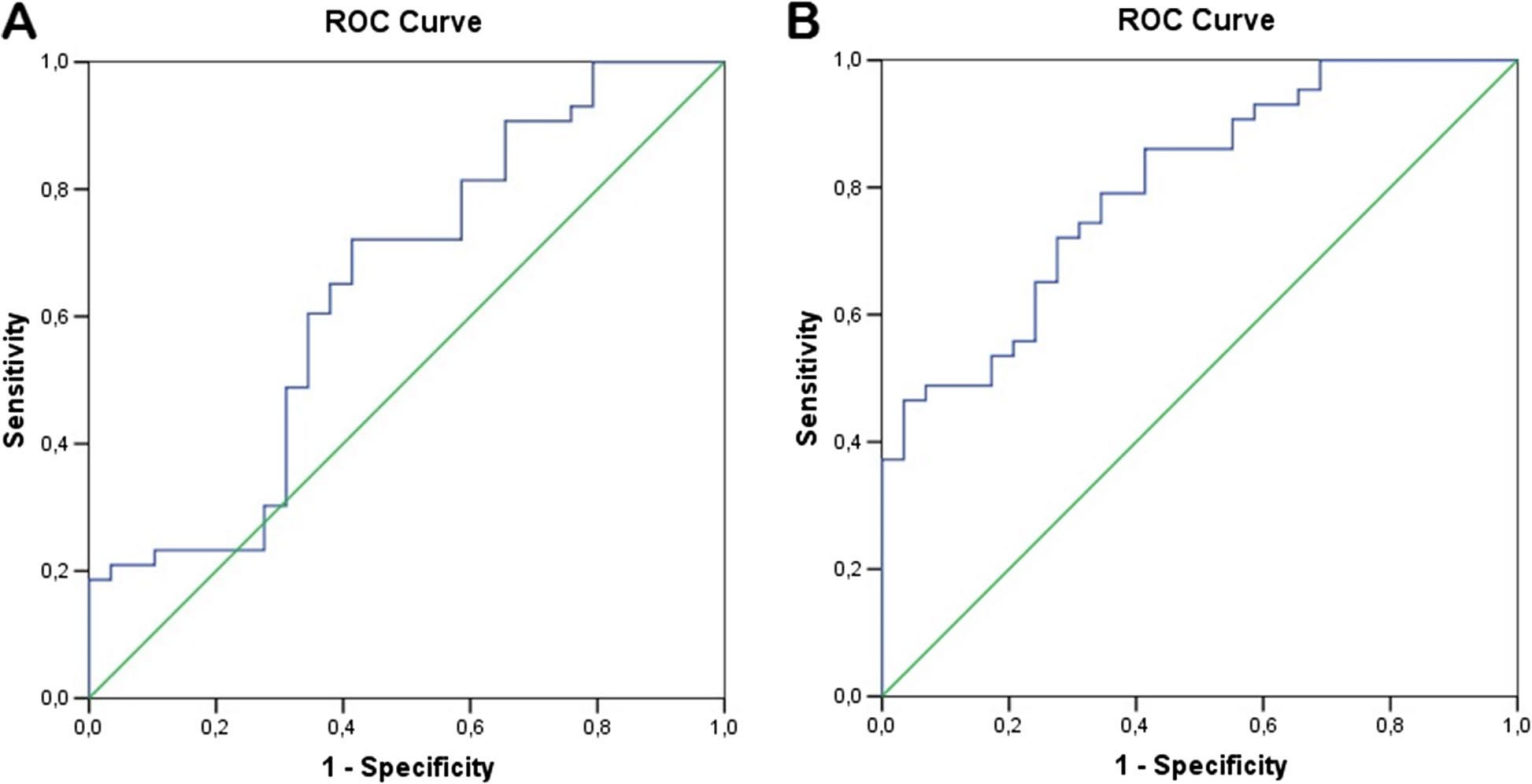
Effect of the prediction model (ROC curve) including: A) baseline FOXP3 and day-15 solCTLA-4, or B) baseline FOXP3- day-15 solCTLA-4-donor age on multivariable logistic regression for the risk for renal dysfunction at one year after transplantation. A) Area under the curve (AUC)=0.645, 95%CI: 0.523-0.754 *p*=0.036. A log cut-off >0.5696 has a higher risk of graft dysfunction after one year from transplantation, with a sensitivity of 72.1% and specificity of 58.6%. B) Area under the curve (AUC)= 0.802, 95%CI=0.691-0.887, *p*<0.001. Older donors (>53 years old) and mRNA CTLA-4 sol and FOXP3 with a log cut-off higher than the value of 0.461 predict an increased risk of graft dysfunction at one year after transplantation, with a sensitivity of 86.1% and specificity of 58.6%

ROC curve analysis obtained by FOXP3 baseline and solCTLA-4 15 days together showed a discriminating power AUC = 0.65 (95%CI = 0.523–0.754, *p* = 0.036), while the addition of donor age in the model improved the AUC to 0.80 (95%CI = 0.691–0.887, *p* < 0.001), proving an accurate diagnostic test (ROC curves comparison *p* = 0.020), Fig. 7. A cut-off value > 0.461 was able to identify patients with graft dysfunction with a sensitivity of 86.0% and a specificity of 58.6%.

## Discussion

The study of urine and peripheral blood biomarkers of aTCMR and immune dysregulation in kidney transplantation is crucial because non-invasive methods are potentially game changers in clinical practice. Tregs have studied in this context for their suppressive capacity and for the prediction of better long-term graft outcomes [2, 12–17]. The role of FOXP3-positive infiltrates in renal allograft biopsies in prolonging organ survival has not yet been clarified, although long-lived grafts demonstrate a substantial presence of FOXP3^+^ Tregs, suggesting their beneficial effect on survival, through a regulatory function [18]. The activity of Tregs modulated by the transcription factor FOXP3 is dependent on the expression of a complex group of proteins, such as CTLA-4 [19]. The profiling of FOXP3 and CTLA-4 isoforms gene expression at baseline and during the first year after kidney transplantation might clarify patterns of immunological activation, for these molecules are strongly implicated in tolerance of transplanted organs. Most of the evidence concerning these candidate biomarkers comes from cross-sectional or case-control studies and the validation of these molecules as immunological biomarkers in longitudinal studies still requires proof of accuracy. The improvement of long-term graft survival is still an important goal in kidney transplantation and the induction of donor-specific tolerance represents the holy grail of solid-organ transplantation.

In our longitudinal prospective and case-control study, we examined in peripheral blood the role played by the FOXP3 and CTLA-4 transcripts as possible biomarkers of clinical outcomes, such as aTCMR, de novo DSA development and renal dysfunction. The analysis was carried out on mRNA transcripts from peripheral blood samples from KTRs monitored up to one year after transplantation, easily available with relatively non-invasive techniques, compared to tissue biomarkers obtained through biopsies. The choice of FOXP3 and CTLA-4 as candidate biomarkers of the immune response to KTR was supported by the evidence of their wide inter-individual variability, both in healthy subjects and in patients on renal replacement therapy (RRT) (Fig. 3, legend).

CTLA-4 is a target gene of FOXP3, which functions as the main Treg regulator, and whose expression levels are critical for the suppressive function of Tregs [20, 21]. CTLA-4 would have a dual function: for conventional Teffs, it would act as a receptor for inhibitory signals, while, on Tregs, as an immune response suppression mediator [22, 23].

We studied the expression of these molecules over a period spanning from before transplantation up to 1 year after transplantation, and we observed that pre-transplant levels of CTLA-4 and FOXP3 were significantly reduced compared to healthy subjects. Accordingly, recent studies and a meta-analysis have suggested that end-stage renal disease (ESRD) and RRT influence immunity by lowering CD4^+^ lymphocytes and Tregs [24, 25]. After transplantation, we documented a robust decrease in both CTLA-4 and FOXP3 expression compared to baseline levels, probably due a combined effect of immunosuppressive treatment and immune response. Our findings are in line with recent results obtained by flow cytometry regarding Tregs 6 months after transplantation [12]. The authors observed a decrease after transplantation in the percentage of both natural thymic Tregs and activated Tregs in peripheral blood. Furthermore, a higher percentage of activated Tregs before transplantation was also a predictive biomarker of long-term graft survival. The fall in biomarkers levels during the first 15 days after transplantation might be a consequence of induction therapy. It is indeed known that these drugs can cause a profound decrease of T and NK cells. In 2015, Krepsova et al. observed a reduction in gene expression of some tolerance–associated transcripts in patients treated also with basiliximab [26]. In the early post-transplant period, CNI-based maintenance therapy could also interfere with FOXP3 transcription by inhibiting IL-2 release.

In our analysis, we observed that the expression of FOXP3 in peripheral blood was lower by the time of aTCMR than in all the other time points. This was a consequence of a recall in the graft of FOXP3^+^ lymphocytes, both Tregs and activated CD4^+^ T cells. Another mechanism is possibly the Treg plasticity, by which a loss of FOXP3 expression would reflect a change from Treg into Teff cells during inflammation for the pro-apoptotic role of FOXP3 [27, 28].

Through the analysis of immunological, demographic and clinical parameters, we detected an association between aTCMR within the first year after transplantation and solCTLA-4 mRNA levels 15 days after transplantation, use of everolimus, and immunosuppression switch, while basiliximab as induction therapy was negatively correlated. Despite the relatively small number of acute rejection cases and, overall, the small sample size of the entire cohort should represent a note of caution for any definitive interpretation, we hypotesize a putative role of early post-transplant levels of solCTLA-4 on the susceptibility to acute rejection, through an influence on T-cell activation. This observation was already reported in autoimmune thyroid diseases, where solCTLA-4 would indirectly act as an enhancer of autoimmune response in the presence of activated intra-thyroid T lymphocytes [29]. In our experience, solCTLA-4 acted as a time-dependent four-fold risk factor for aTCMR and showed a good accuracy of prediction at ROC curve both in the entire KTRs group but mainly in patients treated only with mycophenolate acid. After transplantation, solCTLA-4 could interfere with flCTLA-4 function, stimulate T reactivity and prevent the transduction of inhibitory signals. These data support the role of solCTLA-4 in acute rejection demonstrated at DNA level in one previous genetic study by our group [30], where we showed an increased frequency of solCTLA-4 CT60 A/A genotype in the 3′ untranslated region in patients experiencing acute rejection. This genotype predisposes to higher release of solCTLA-4 and it has been also emphasised in graft-versus-host and autoimmune diseases [31–35]. There is growing experimental and clinical evidence that soluble isoforms play an important role in establishing and maintaining peripheral tolerance [36]. Despite the initial observation that solCTLA-4 is mainly produced by *resting* T cells [37], recent studies have clarified that solCTLA-4 release can rise during antigenic responses, and that this phenomenon modulates immune responses [26, 38–40].

In addition, the protective influence of basiliximab compared with rATG has already been observed by other groups that evidenced, in the early post-transplant period, a higher ratio CD4^+^FOXP3^+^ Tregs to effector T cells and an inferior incidence of rejection in KTRs treated with basiliximab [26, 41].

Interestingly, we found out a negative correlation between the expression of baseline solCTLA-4 and de novo DSA production after transplantation. This association could be interpreted by considering the control that Tregs exert, through the CTLA-4 co-receptor, on the expansion of follicular T cells and the humoral immunity. Memory B cells are important in alloreactivity in kidney transplantation and B cells are involved in aTCMR as well as in chronic antibody-mediated rejection with DSA produced by B cells [42–47].

FOXP3 is involved in the regulation of Tregs development and function, and it is considered a biomarker of kidney allograft tolerance. Previous studies, based on urinary FOXP3 mRNA of KTRs, showed higher levels in patients experiencing acute rejection compared to patients with chronic rejection or subjects with stable function [16, 48]. In our study, FOXP3 mRNA levels in peripheral blood were not associated with aTCMR at regression analysis but, at the time of rejection, we recorded the lowest level of transcription of the entire follow-up. However, median expression levels in aTCMR-positive patients showed gradual expression increment up to 1 year after transplantation, while on the contrary in negative-aTCMR KTRs they first decreased until 60 days after transplantation and then increased.

In addition, in recipients with impaired graft function 1 year after transplantation a higher expression of pre-transplant FOXP3 and day-15 solCTLA-4 emerged. This finding suggests that these molecules might work as prognostic biomarkers, whose prediction power is increased when considering donor age. Older donors (> 53 years) with day-15 solCTLA-4 and baseline FOXP3 mRNA log> 0.461 have an increased risk of graft dysfunction at 1 year post-transplantation.

This association between day-15 solCTLA-4 and renal dysfunction might originate from the influence of Tregs on memory CD8^+^CD28^−^ Teff, which has been recently implied in allograft dysfunction [49, 50]. It is known that ESRD patients harbour a heterogeneous population of CD3^+^CD8^+^CD28^−^ cells with immunomodulatory but also cytotoxic characteristics to a greater extent than healthy subjects do, and which expands after transplantation [51].

The study presents some limitations. First, a relative heterogeneity exists in terms of immunosuppressive therapy adopted in the cohort. Such a phenomenon should be connected with the long period of enrollment. However, this study represents a monocenter experience, therefore limiting the potential biases derived from different managements and clinical approaches. Overall, the decision to use different immunosuppressive regimens derived from several aspects, like specific clinical necessities (using mTOR inhibitors in patients with previous history of cancer), specific immunological background (pre-transplant PRA value), or protocols of research. In all the cases, the best immunosuppressive regimens were used following the available literature.

As previously reported, another limit to consider is the sample size of the event of interest, namely the aTCMR. Unfortunately, this is the principal limit of studies focused on investigating relatively uncommon events, like aTCMR is. In this observational study we enrolled 125 consecutive kidney transplant patients, reporting 11 (8.8%) and 16 (12.8%) aTCMR cases during the first year and duting the overall period of the study. From a clinical point of view, the aTCMR frequency is in line with previous clinical experiences. We understand that the study’s small sample size can unfortunately impair our ability to construct solid diagnostic models. However, on the other side we should underline that the Treg markers studied in the present analysis have been mainly investigated in cross-sectional or case-control studies, and that their impact in longitudinal studies still requires proof of accuracy. Therefore, we think that, although limited by the numbers, our experience should be of relevance.

## Conclusions

The molecular study of clinically relevant Treg markers in peripheral blood of KTRs suggested a dual immunological role for the solCTLA-4 molecule, which might predict a susceptibility to cellular acute rejection and/or graft dysfunction, but, on the other hand, a protection towards de novo DSA response. Furthermore, FOXP3 monitoring could be useful as indicator of acute rejection for the effect of Treg plasticity, reflective of a change from Treg into Teff cells during inflammation for the pro-apoptotic role of FOXP3, and contribute with solCTLA-4 to increased risk of graft dysfunction at one year post-transplantation.

While the potential of the soluble CTLA-4 isoform as a therapeutic target will need a wider case history and a confirmation of its role, verifying the levels of the protein in the serum or plasma, the dosage of the blood levels of these two molecules, and mainly of the solCTLA-4, could put forward as a candidate non-invasive biomarker of cellular and humoral alloreactivity in clinical transplantation. mRNA levels of Treg-associated genes in peripheral blood might help shape immunosuppression, tailor monitoring and achieve a better long-term clinical course of kidney transplantation in the wake of “precision medicine”.

## Supplementary Information

**Additional file 1. **Figure S1 – Comparison of mRNA levels of FOXP3 (mean±SD) monitored during the first post-transplant year in patients treated with mycophenolic acid compared to everolimus (RT-PCR reference: healthy controls). There were no significant difference between recipients on everolimus and recipients on mycophenolic acid for the whole duration of the monitoring. The 12 patients on everolimus showed a similar consistent reduction after 15 days from transplantation compared to baseline levels (RQ=0.722, IQR=0.375-0.946 vs. RQ=1.521, IQR=1.073-2.549, p=0.004). Therefore, the cases with everolimus experienced a gradual increase at 60 days, (RQ=0.806, IQR=0.428-1.463, p=0.005) and at one year (RQ=1.343, IQR=0.461-1.574, one-way ANOVA p=0.002).

**Additional file 2.**

**Additional file 3.**

**Additional file 4.**

## Data Availability

Data from this study are available in the article and its supplementary information files or by the corresponding upon plausible request.

## Abbreviations

aTCMR: Acute T-cell-mediated rejection
APC: Antigen-presenting cell
AUC: Area-under-the-curve
β2M: β2 Microglobulin
CI: 95% Confidence interval
CNI: Calcineurin inhibitor
CTLA-4: Cytotoxic T-Lymphocyte Antigen 4
DSA: Donor-specific antibody
eGFR: Estimated glomerular filtration rate
ESRD: End-stage renal disease
flCTLA-4: Full-length CTLA-4
FOXP3: Forkhead box P3
GADPH: Glyceraldehyde 3-phosphate dehydrogenase
HR: Hazard ratio
IQR: Interquartile ranges
KTR: Kidney transplant recipient
mTORi: Mammalian-target-of-rapamycin inhibitor
OR: Odds ratio
ROC: Receiver-operating characteristic
RQ: Relative quantification
RRT: Renal replacement therapy
(RT-)PCR: (Real-time) polymerase-chain reaction
solCTLA-4: Soluble CTLA-4
Teff: Effector T cell
Tfh: Follicular helper T cell
Tfr: Follicular regulatory T cell
Treg: Regulatory T cell
