# Supplementary figures and images for: Longitudinal monitoring of mRNA levels of regulatory T cell biomarkers by using non-invasive strategies to predict outcome in renal transplantation
Source: BMC Nephrol. 2022 Feb 2;23:51. doi: 10.1186/s12882-021-02608-3 (PMC8809010; doi:10.1186/s12882-021-02608-3)

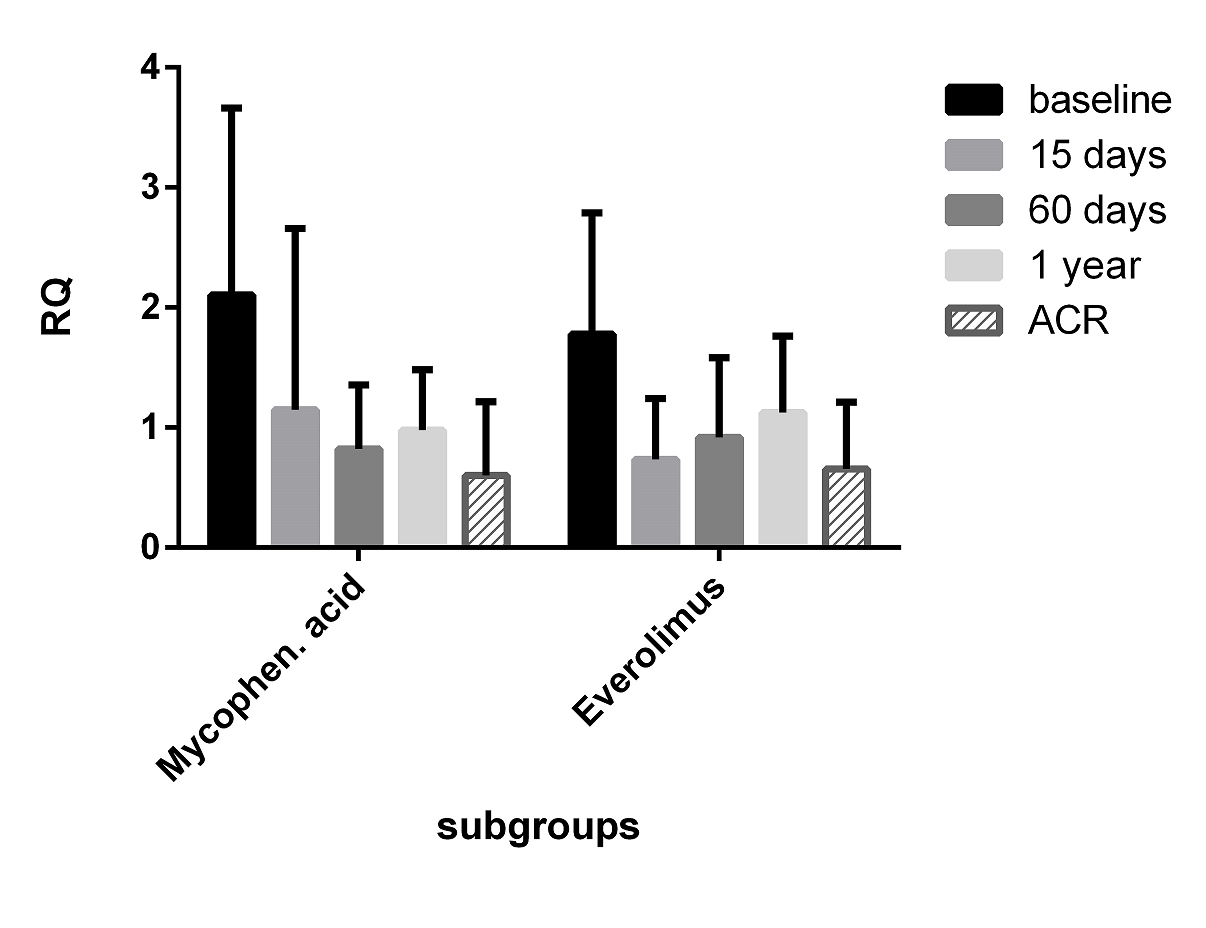

Supplement: Supplementary file 1 — Additional file 1. Figure S1 – Comparison of mRNA levels of FOXP3 (mean±SD) monitored during the first post-transplant year in patients treated with mycophenolic acid compared to everolimus (RT-PCR reference: healthy controls). There were no significant difference between recipients on everolimus and recipients on mycophenolic acid for the whole duration of the monitoring. The 12 patients on everolimus showed a similar consistent reduction after 15 days from transplantation compared to baseline levels (RQ=0.722, IQR=0.375-0.946 vs. RQ=1.521, IQR=1.073-2.549, p=0.004). Therefore, the cases with everolimus experienced a gradual increase at 60 days, (RQ=0.806, IQR=0.428-1.463, p=0.005) and at one year (RQ=1.343, IQR=0.461-1.574, one-way ANOVA p=0.002). [file 12882_2021_2608_MOESM1_ESM.tif]
