# Supplementary material for: Longitudinal monitoring of mRNA levels of regulatory T cell biomarkers by using non-invasive strategies to predict outcome in renal transplantation
Source: BMC Nephrol. 2022 Feb 2;23:51. doi: 10.1186/s12882-021-02608-3 (PMC8809010; doi:10.1186/s12882-021-02608-3)
Supplement: Supplementary file 2 — Additional file 2. [file 12882_2021_2608_MOESM2_ESM.docx]

**Table S1**. **Univariable and multivariable logistic regression for the risk of *de novo* DSA development after 1 year from kidney transplantation.**

| **Variable** | **Univariable analysis** | | | **Multivariable analysis^a^**  -2log likelihood: 53.237 | | |
| --- | --- | --- | --- | --- | --- | --- |
|  | **OR** | **95% CI** | **P** | **AOR** | **95% CI** | **P** |
| **Baseline** |  |  |  |  |  |  |
| *Full-lenght CTLA4 | 0.385 | 0.103-1.436 | 0.155 | — | — | — |
| ***Soluble CTLA4*** | **0.284** | 0.085-0.8956 | **0.042** | **0.110** | **0.023-0.537** | **0.006** |
| FOXP3 | 2.530 | 0.482-13.293 | 0.273 | — | — | — |
| **At 15 days** |  |  |  |  |  |  |
| Full-lenght CTLA4 | 0.624 | 0.211-1.844 | 0.394 | — | — | — |
| ***Soluble CTLA4*** | **0.325** | 0.116-0.911 | **0.033** | — | — | — |
| FOXP3 | 1.246 | 0.392-3.962 | 0.710 | — | — | — |
| **At 60 days** |  |  |  |  |  |  |
| Full-lenght CTLA4 | 0.202 | 0.038-1.079 | 0.061 | — | — | — |
| ***Soluble CTLA4*** | **0.167** | 0.044-0.636 | **0.009** | — | — | — |
| FOXP3 | 1.272 | 0.312-5.198 | 0.737 | — | — | — |
| **At one year** |  |  |  |  |  |  |
| Full-lenght CTLA4 | 1.030 | 0.112-9.468 | 0.979 | — | — | — |
| Soluble CTLA4 | 4.207 | 0.339-52.153 | 0.263 | — | — | — |
| FOXP3 | 7.493 | 0.286-196.103 | 0.227 | — | — | — |
| Recipient age | 0.966 | 0.926-1.009 | 0.122 | — | — | — |
| Recipient gender | 1.043 | 0.331-3.291 | 0.943 | — | — | — |
| Donor age | 1.004 | 0.972-1.037 | 0.790 | — | — | — |
| Donor gender | 0.476 | 0.169-1.341 | 0.160 | — | — | — |
| Type of donor | 2.627 | 0.406-16.991 | 0.310 | — | — | — |
| Previous transplantation | 0.722 | 0.079-6.581 | 0.773 | — | — | — |
| HLA mismatch | 0.833 | 0.555-1.248 | 0.375 | — | — | — |
| **cRF (first class+second class)** | **19.059** | 2.411-150.661 | **0.005** | — | — | — |
| CIT | 1.000 | 0.998-1.002 | 0.906 | — | — | — |
| WIT | 0.996 | 0.957-1.038 | 0.859 | — | — | — |
| Type of renal replacement therapy | 1.034 | 0.259-4.140 | 0.962 | — | — | — |
| Dialysis time (groups) | 1.459 | 0.818-2.602 | 0.200 | — | — | — |
| CMV reactivation | 0.503 | 0.132-1.924 | 0.316 | — | — | — |
| Type of induction^b^ | 1.529 | 0.273-8.580 | 0.629 | — | — | — |
| Use of cyclosporine^c^ | 1.006 | 0.250-4.042 | 0.994 | — | — | — |
| Use of everolimus | 2.067 | 0.549-7.784 | 0.283 | — | — | — |
| Immunosuppression change | 1.429 | 0.440-4.636 | 0.553 | — | — | — |
| DGF | 1.050 | 0.366-3.008 | 0.928 | — | — | — |
| ^a^ Model summary: χ2(1)=9.482, p=0.002; Nagelkerke R2=0.225; Hosmer and Lemeshow χ2 test=6.506, p=0.591. Covariates initially introduced in the multivariable model and then elided were: soluble CTLA4 at 15 days, soluble CTLA4 at 60 days. *mRNA expression in log2-DCT. | | | | | | |
