# Supplementary material for: Longitudinal monitoring of mRNA levels of regulatory T cell biomarkers by using non-invasive strategies to predict outcome in renal transplantation
Source: BMC Nephrol. 2022 Feb 2;23:51. doi: 10.1186/s12882-021-02608-3 (PMC8809010; doi:10.1186/s12882-021-02608-3)
Supplement: Supplementary file 3 — Additional file 3. [file 12882_2021_2608_MOESM3_ESM.docx]

**Table S2. Univariable and multivariable logistic regression for the risk of *de novo* DSA development within 2 years from kidney transplantation.**

| **Variable** | **Univariable analysis** | | | **Multivariable analysis^a^**  -2log likelihood: 53.789 | | |
| --- | --- | --- | --- | --- | --- | --- |
|  | **OR** | **95% CI** | **P** | **AOR** | **95% CI** | **P** |
| **Baseline** |  |  |  |  |  |  |
| *Full-lenght CTLA4 | 0.459 | 0.131-1.611 | 0.224 | — | — | — |
| ***Soluble CTLA4*** | **0.239** | 0.071-0.801 | **0.020** | **0.086** | **0.016-0.453** | **0.004** |
| FOXP3 | 1.914 | 0.431-8.492 | 0.393 | — | — | — |
| **At 15 days** |  |  |  |  |  |  |
| Full-lenght CTLA4 | 0.710 | 0.248-2.029 | 0.522 | — | — | — |
| ***Soluble CTLA4*** | **0.282** | 0.100-0.794 | **0.017** | — | — | — |
| FOXP3 | 1.540 | 0.489-4.850 | 0.461 | — | — | — |
| **At 60 days** |  |  |  |  |  |  |
| Full-lenght CTLA4 | 0.287 | 0.059-1.405 | 0.124 | — | — | — |
| ***Soluble CTLA4*** | **0.150** | 0.039-0.572 | **0.005** | — | — | — |
| FOXP3 | 1.110 | 0.287-4.301 | 0.880 | — | — | — |
| **At one year** |  |  |  |  |  |  |
| Full-lenght CTLA4 | 1.184 | 0.184-9.711 | 0.875 | — | — | — |
| Soluble CTLA4 | 2.230 | 0.275-19.761 | 0.438 | — | — | — |
| FOXP3 | 2.926 | 0.227-37.640 | 0.410 | — | — | — |
| Recipient age | 0.970 | 0.930-1.011 | 0.144 | — | — | — |
| Recipient gender | 1.346 | 0.435-4.165 | 0.606 | — | — | — |
| Donor age | 1.000 | 0.970-1.031 | 0.994 | — | — | — |
| Donor gender | 0.396 | 0.147-1.066 | 0.067 | — | — | — |
| Type of donor | 5.132 | 0.798-32.990 | 0.085 | — | — | — |
| Previous transplantation | 0.590 | 0.065-5.347 | 0.639 | — | — | — |
| HLA mismatch | 0.937 | 0.640-1.372 | 0.738 | — | — | — |
| cRF (first class) | 0.000 | 0.000 | 0.998 | — | — | — |
| cRF (second class) | 0.075 | 0.010-0.592 | 0.014 | — | — | — |
| CIT | 0.999 | 0.998-1.001 | 0.553 | — | — | — |
| WIT | 1.002 | 0.965-1.041 | 0.915 | — | — | — |
| Type of renal replacement therapy | 1.309 | 0.332-5.165 | 0.701 | — | — | — |
| Dialysis time | 1.003 | 0.989-1.016 | 0.695 | — | — | — |
| CMV reactivation | 0.605 | 0.180-2.030 | 0.416 | — | — | — |
| Type of induction^b^ | 1.240 | 0.223-6.894 | 0.806 | — | — | — |
| Use of cyclosporine^c^ | 0.804 | 0.203-3.191 | 0.756 | — | — | — |
| Use of everolimus | 1.639 | 0.442-6.081 | 0.460 | — | — | — |
| Immunosuppression change | 1.558 | 0.510-4.758 | 0.437 | — | — | — |
| DGF | 0.784 | 0.281-2.184 | 0.642 | — | — | — |
| ^a^ Model summary: χ2(1)=11.404, p=0.001; Nagelkerke R^2^=0.261; Hosmer and Lemeshow χ2 test=6.687, p=0.571. Covariates initially introduced in the multivariable model and then elided were: soluble CTLA4 at 15 days, soluble CTLA4 at 60 days. *mRNA expression in log2^-DCT^. Abbreviations: OR, odds ratio; CI, confidence intervals; AOR, adjusted OR; BMI, body mass index; HLA, human leukocyte antigens; CIT, cold ischemia time; WIT, warm ischemia time. | | | | | | |
