# Supplementary material for: Longitudinal monitoring of mRNA levels of regulatory T cell biomarkers by using non-invasive strategies to predict outcome in renal transplantation
Source: BMC Nephrol. 2022 Feb 2;23:51. doi: 10.1186/s12882-021-02608-3 (PMC8809010; doi:10.1186/s12882-021-02608-3)
Supplement: Supplementary file 4 — Additional file 4. [file 12882_2021_2608_MOESM4_ESM.docx]

**Table S3. Univariable and multivariable logistic regression for the risk of graft dysfunction within two years (n=86) from kidney transplantation.**

| **Variables** | **Univariable analysis** | | | **Multivariable analysis^a^**  -2log likelihood: 83.404 | | |
| --- | --- | --- | --- | --- | --- | --- |
|  | **OR** | **95% CI** | **P** | **AOR** | **95% CI** | **P** |
| **Baseline** |  |  |  |  |  |  |
| Full-length CTLA4 | 1.845 | 0.583-5.837 | 0.297 | — | — | — |
| Soluble CTLA4 | 1.536 | 0.556-4.396 | 0.397 | — | — | — |
| FOXP3 | 1.888 | 0.559-6.374 | 0.306 | — | — | — |
| **At 15 days** |  |  |  |  |  |  |
| Full-length CTLA4 | 1.961 | 0.720-5.336 | 0.187 | — | — | — |
| Soluble CTLA4 | 2.195 | 0.863-5.582 | 0.099 | — | — | — |
| FOXP3 | 1.577 | 0.586-4.240 | 0.367 | — | — | — |
| **At 60 days** |  |  |  |  |  |  |
| Full-length CTLA4 | 1.808 | 0.483-6.773 | 0.379 | — | — | — |
| Soluble CTLA4 | 1.172 | 0.386-3.556 | 0.779 | — | — | — |
| FOXP3 | 1.127 | 0.377-3.373 | 0.831 | — | — | — |
| **At one year** |  |  |  |  |  |  |
| Full-length CTLA4 | 1.356 | 0.163-11.318 | 0.778 | — | — | — |
| Soluble CTLA4 | 2.574 | 0.302-21.913 | 0.387 | — | — | — |
| FOXP3 | 4.381 | 0.261-73.437 | 0.304 | — | — | — |
| **Recipient age** | 1.068 | 1.021-1.116 | **0.004** | — | — | — |
| Recipient gender | 1.023 | 0.392-2.665 | 0.963 | — | — | — |
| **Donor age** | 1.070 | 1.032-1.109 | **<0.001** | **1.063** | 1.023-1.105 | **0.002** |
| Donor gender | 0.529 | 0.213-1.314 | 0.170 | — | — | — |
| Type of donor | 0.701 | 0.111-4.422 | 0.705 | — | — | — |
| Previous transplantation | 1.658 | 0.263-10.462 | 0.591 | — | — | — |
| HLA mismatch | 1.198 | 0.853-1.682 | 0.296 | — | — | — |
| cRF (first-second class) | 0.768 | 0.324-1.822 | 0.549 | — | — | — |
| CIT | 1.000 | 0.999-1.002 | 0.674 | — | — | — |
| WIT | 1.027 | 0.992-1.064 | 0.130 | — | — | — |
| Type of renal replacement therapy | 1.796 | 0.586-5.502 | 0.305 | — | — | — |
| Dialysis time | 1.048 | 0.342-3.207 | 0.935 | — | — | — |
| CMV reactivation | 1.247 | 0.465-3.345 | 0.662 | — | — | — |
| Type of induction^b^ | 0.400 | 0.073-2.187 | 0.290 | — | — | — |
| Use of cyclosporine^c^ | 1.535 | 0.483-1.585 | 0.468 | — | — | — |
| Use of everolimus | 1.337 | 0.3754.769 | 0.654 | — | — | — |
| **Immunosuppression change** | 3.621 | 1.158-11.323 | **0.027** | — | — | — |
| DGF | 1.707 | 0.685-4.253 | 0.251 | — | — | — |
| Development of DSA | 1.247 | 0.465-3.345 | 0.662 | — | — | — |
| Proteinuria at one year (mg/l) | 1.002 | 0.999-1.005 | 0.243 | — | — | — |
| ^a^ Model summary: χ^2^(1)=12.236 , p=0.000; Nagelkerke R^2^=0.217; Hosmer and Lemeshow χ^2^ test=11.898, p=0.156. Covariates initially introduced in the multivariable model and then elided were: recipient age, cRF class I and DGF; ^b^ use of anti-thymocyte globulins vs. use of anti-IL2 receptor-α monoclonal antibodies; ^c^ vs. tacrolimus. Abbreviations: OR, odds ratio; CI, confidence intervals; AOR, adjusted OR; BMI, body mass index; HLA, human leukocyte antigens; CIT, cold ischemia time; WIT, warm ischemia time. | | | | | | |
